# Nanoemulsions of Sicilian lemon and wild orange essential oils, using soy lecithin as a surfactant, preserve the acrosome of ram sperm post-thawing

**DOI:** 10.1590/1984-3143-AR2024-0110

**Published:** 2025-10-17

**Authors:** Aline Saraiva de Oliveira, Lúcia Cristina Pereira Arruda, Gustavo de Oliveira Alves Pinto, Amanda Rodrigues dos Santos Magnabosco, André Lucas Correa de Andrade, Pabyton Gonçalves Cadena, Maria Madalena Pessoa Guerra

**Affiliations:** 1 Laboratório de Andrologia – ANDROLAB, Departamento de Medicina Veterinária, Universidade Federal Rural de Pernambuco, Recife, PE, Brasil; 2 Laboratório de Ecofisiologia e Comportamento Animal – LECA, Departamento de Morfologia e Fisiologia Animal, Universidade Federal Rural de Pernambuco, Recife, PE, Brasil

**Keywords:** semen cryopreservation, nanotechnology, limonene

## Abstract

The objective of this study was to evaluate the effect of adding Sicilian lemon and wild orange essential oil nanoemulsion, using soy lecithin as a surfactant, to ram semen freezing extender. The nanoemulsions were prepared by high-energy emulsification method using soy lecithin (5%) as a surfactant. The organoleptic and physicochemical characteristics were evaluated. Semen samples (n = 7) obtained from adult rams (n = 6) were frozen in a Tris-egg yolk extender supplemented with Sicilian lemon or wild orange nanoemulsion at different concentrations (0.0%, 1.5%, 2.5%, and 3.5%). After thawing (37^o^C, 30 s), the samples were evaluated for kinematics, plasma and acrosomal membrane integrity, and mitochondrial membrane potential. Visually, the nanoemulsions of Sicilian lemon or wild orange essential oil appeared homogeneous, fluid, opaque, without lumps, odorless, and colored, immediately after preparation (0 h) and after thermal stress (24 h). The physicochemical characterization of the nanoemulsions showed vesicles with average sizes < 220.00 nm, polydispersity index < 0.30, and zeta potential of -59.00 mV. Semen samples from the groups treated with Sicilian lemon (1.5%, 2.5%, and 3.5%) or wild orange (1.5%, 2.5%, and 3.5%) nanoemulsions did not differ (P ≤ 0.05) in terms of kinematics, plasma membrane integrity, and mitochondrial membrane potential when compared to the control group. However, the groups treated with Sicilian lemon (2.5% and 3.5%) and wild orange (1.5%, 2.5%, and 3.5%) nanoemulsions had a higher percentage (P ≤ 0.05) of cells with intact acrosomes when compared to the control group. It can be concluded that nanoemulsions of essential oils of Sicilian lemon (2.5% and 3.5%) and wild orange (1.5%, 2.5%, and 3.5%), using soy lecithin (5%) as a surfactant, can be used as additives to the Tris-egg yolk extender for ram semen freezing due to their ability to preserve the acrosome post-thawing.

## Introduction

Semen cryopreservation is a reproductive biotechnique that enables the formation of a germplasm bank and allows semen to be stored indefinitely (Yánez-Ortiz et al., 2022). This process reduces costs related to broodstock acquisition (Lv et al., 2019). When combined with genetic improvement programs, it supports the dissemination of genetically superior material (Arando et al., 2017). It also facilitates the use of other reproductive biotechniques, such as artificial insemination (Lv et al., 2019), ultimately improving the reproductive and productive performance of herds.

Despite being a widely used biotechnique, the number of viable cells after the freezing and thawing process remains variable. This is because semen cryopreservation causes structural and molecular changes in the cells, leading to a reduction in their fertilizing capacity and embryonic development (Yánez-Ortiz et al., 2022). These changes are attributed to temperature variation, oxidative damage, osmotic stress, and cell membrane damage, among other factors (Yeste, 2017).

Considering that semen extenders play an important role in the freezing process by protecting sperm and minimizing damage, it is essential to understand the functionality of their constituents and to optimize them (Mokhtassi-Bidgoli et al., 2023). Given this, and considering the cytotoxicity of cryoprotectants used in freezing extenders (Yeste, 2017), it is necessary to combine the biotechnique of semen cryopreservation with new technologies, such as nanotechnology. This approach aims to develop more effective extenders that preserve a greater number of viable sperm cells after freezing while maintaining their fertilizing capacity.

Nanotechnology is based on the production and characterization of materials at the atomic scale, so-called nanomaterials (Hulla et al., 2015). Specifically, nanoemulsions are defined as dispersed nanostructured systems composed of two immiscible liquids, usually water and oil, which are homogenized and stabilized by the addition of emulsifiers (Singh et al., 2017).

Among the oils that can be used in the formulation of nanoemulsions, essential oils stand out. These are volatile compounds derived from the secondary metabolism of aromatic plants (Osanloo et al., 2020) and are known for their antioxidant, antimicrobial, and anti-inflammatory activities (Sitarek et al., 2017). Citrus essential oils—such as wild orange, tangerine, and lemon (Palazzolo et al., 2013)—consist of 85% to 99% volatile compounds and 1% to 15% non-volatile compounds. The volatile fraction is primarily composed of terpenes, including monoterpenes and sesquiterpenes (Mehl et al., 2014), with limonene (a monoterpene) being the predominant constituent (Fisher and Phillips, 2008; Santos et al., 2023).

Studies have demonstrated the antioxidant potential of limonene (Anandakumar et al., 2021), which inhibits malonaldehyde production, disrupts the lipid oxidation cycle (Wei and Shibamoto, 2007), and eliminates oxidants (Davicino et al., 2009). In sperm cells, both S-(−) limonene and R-(+) limonene, when added to the cryopreservation extender of bull semen, show no deleterious effects on the cells (Branco et al., 2016a, 2016b). However, S-(−) limonene significantly increases the linearity of these gametes (Branco et al., 2016a).

Despite studies investigating the antioxidant activity of limonene in various cell types, there are no reports on the activity of limonene-rich essential oils—specifically those obtained from Sicilian lemon and wild orange—in sperm cells when used in association with nanostructured systems. In light of this, the objective of the present study was to evaluate the effect of adding nanoemulsions of Sicilian lemon and wild orange essential oils, formulated with soy lecithin (5%) as a surfactant, to the ram semen freeze extender.

## Methods

### Reagents

The essential oils of Sicilian lemon (lot# 2202541, Citrus limon, Chemotype Limonene) and wild orange (lot# 2202753, Citrus sinensis, Chemotype Limonene) were purchased from doTERRA (Barueri, SP, Brazil) and were previously characterized by gas chromatography according to the supplier (Table 1). All reagents used were purchased from Sigma-Aldrich (St. Louis, MO, USA), except buffered salt phosphate (PBS), purchased from Gibco® (Life Technologies, Carlsbad, CA, USA). Stock solutions of fluorochromes were prepared as follows: Propidium iodide (IP; 25mg/mL), JC-1 (5mg/mL), and FITC-conjugated to Peanut agglutinin (FITC-PNA: 1mg/mL) in PBS, while the working solutions JC-1 (153μM) and carboxyfluorescein diacetate (DCF; 0.46 mg/mL) were prepared in Dimethyl sulfoxide (DMSO), and FITC-PNA (0.04mg/mL) and IP (0.5mg/mL) were prepared in PBS. All solutions were stored at -20 °C until the time of use.

**Table 1:** Chemical composition of lemon (*Citrus limon*) and wild orange (*Citrus sinensis*) essential oil. Data (gas chromatography) was provided by the supplier.

**Essential oil**	**Compounds**	**Value (%)**
**Sicilian lemon**	Limonene	64.96
	β-Pinene	11.56
	Ω- Terpinene	9.75
	Sabinene	2.05
	α-Pinene	2.02
	Myrcene	1.62
	Geranial	1.59
	Other	6.45
**Wild Orange**	Limonene	93.77
	Myrcene	2.25
	Other	3.98

### Production of nanoemulsions

The oil/water (O/W) nanoemulsions were produced using the high-energy emulsification method through magnetic stirring (Santos-Magnabosco et al., 2022). Briefly, the aqueous and oil phases were prepared separately. The aqueous phase was made by hydrating soy lecithin (phosphatidylcholine), used as a surfactant, in ultrapure water at a concentration of 5% (v/v). Hydration was carried out in a water bath at 37°C for 60 minutes, followed by homogenization on a magnetic stirrer at 7 *g* for 20 minutes. After a homogeneous dispersion was obtained, the essential oil of Sicilian lemon or wild orange—used as the oil phase at a concentration of 1% (v/v)—was slowly added at a rate of 0.05 mL per second under continuous stirring at 7 *g* for 5 minutes. The resulting emulsions were then kept under stirring at 75 *g* for 24 hours at room temperature (25°C ± 2°C) to obtain the final O/W nanoemulsion.

### Macroscopic physical analysis

The organoleptic characteristics of the nanoemulsions were evaluated immediately after formulation, based on the following criteria (ANVISA, 2004; Damasceno et al., 2016): appearance - dispersion phase: homogeneous (HM) or heterogeneous (HT); appearance – consistency: fluid (FL), slightly thick (PE), thick (ES) or very thick (ME); appearance – opacity: translucent (TR), slightly opaque (PO) or opaque (OP); appearance – lumps: present (PR) or absent (AT); odor: present (PR) or absent (AT); color: present (PR) or absent (AT).

A stability test of the nanoemulsions was also carried out. The nanoemulsions were stored in sterile containers with a headspace equivalent to one-third of the total volume, allowing for gas exchange. Immediately after preparation, samples were subjected to centrifugation at 1210 × *g* for 30 minutes (Kacil CE-01 Centrifuge; Recife, Pernambuco, Brazil), followed by an analysis of appearance—specifically, evaluation of the dispersion phase as either homogeneous (HM) or heterogeneous (HT). Samples were also subjected to thermal stress and evaluated after 24 hours of cooling (5°C ± 2°C), freezing (−20°C ± 2°C), and heating (37°C, 40°C, and 50°C ± 2°C). After these thermal stress tests, the nanoemulsions were assessed at room temperature (25°C ± 2°C) for macroscopic physical characteristics, and the results were compared with those obtained immediately after formulation (Santos-Magnabosco et al., 2022).

### Physicochemical characterization

The nanoemulsions were previously diluted (50×) in ultrapure water to reduce turbidity. The average size (nm), polydispersity index (PDI), and zeta potential (ζ, mV) of the oil vesicles were evaluated using the standard photon correlation spectroscopy technique, set at 90 degrees and 25°C, with a Zetasizer Nano ZS system (Malvern Panalytical, Malvern, UK) (Cadena et al., 2013). All measurements were performed in triplicate. After formulation, the nanoemulsions were stored under refrigeration (5°C) and protected from light until use as a supplement to the Tris-egg yolk diluent.

### Semen extender

The semen freezing extender Tris–egg yolk was composed of 3.605 g of Tris, 2.024 g of citric acid, 1.188 g of fructose, 100 mL of distilled water, 20% egg yolk, 5% glycerol, 100 IU of penicillin, and 50 mg of streptomycin, with a pH of 7.2. Prior to the addition of glycerol and antimicrobials, the diluent was double-centrifuged at 5,500 × *g* for 1 hour. The supernatant and pellet were then discarded, using only the contents of the intermediate region. Afterward, the diluent was filtered through a 0.45-µm membrane and aliquoted to form groups supplemented with nanoemulsions at concentrations of 0.0%, 1.5%, 2.5%, and 3.5%. Following preparation, the samples were stored at −20°C until use, at which point they were thawed in a water bath at 37°C.

### Animals and semen collection

All experimental procedures involving animals were approved by the Ethics Committee for the Use of Animals (CEUA) of the Federal Rural University of Pernambuco (license 6719080520, ID 000304). Six sexually mature Dorper rams with a history of good fertility were used. The animals were housed on a rural property located in the municipality of Coruripe, Alagoas (−10.124067892605936, −36.11322829036088), kept in an intensive confinement system, and fed a balanced diet based on Tifton grass hay and commercial pelleted feed. They also had access to mineral supplementation and water *ad libitum*. Ejaculates were collected every 48 hours using an artificial vagina, with a female present as a mannequin. Seven samples were collected per animal, totaling 42 ejaculates.

### Semen assessment

Initially, the semen samples were subjectively evaluated using a phase contrast microscope (100×; Olympus, Tokyo, Japan) for the parameters of mass movement (0–5), vigor (0–5), and total motility (0%–100%). Samples meeting the minimum criteria—mass movement of ≥3, vigor of ≥3, and total motility of ≥70%—were approved and included in the formation of the seminal pool (n = 7). The sperm concentration was then determined by cell counting in a Neubauer chamber, using a 1:400 dilution.

### Semen dilution and freezing

Each pool was diluted to a concentration of 200 × 10^6^ spermatozoa/mL in Tris–egg yolk, with or without the addition of Sicilian lemon (*Citrus limon*) or wild orange (*Citrus sinensis*) essential oil nanoemulsions. This resulted in the formation of four experimental groups for each nanoemulsion (0.0%, 1.5%, 2.5%, and 3.5%). Subsequently, all samples were filled into 0.25-mL straws and frozen using an automated system (TK 3000^®^; TK Tecnologia em Congelação Ltda., Uberaba, Minas Gerais, Brazil). For refrigeration, the cooling curve followed a rate of −0.25°C/min until reaching 5°C, where the samples remained for 120 minutes (stabilization time). The freezing curve then proceeded at −20°C/min until −120°C, at which point all straws were immersed and stored in liquid nitrogen (−196°C).

### Thawing and semen analysis

For analysis, two straws from each experimental group were thawed in a water bath (37°C for 30 seconds) and incubated at 37°C for 5 minutes. Subsequently, the semen samples were evaluated for sperm kinematics using computer-assisted sperm analysis, as well as for plasma membrane integrity, acrosomal membrane integrity, and mitochondrial membrane potential, using epifluorescence microscopy (Carl Zeiss, Göttingen, Germany).

#### Automated sperm analysis system (CASA)

For the analysis of sperm kinematics, an aliquot (10 μL) of semen was diluted to a concentration of 50 x 10^6^ in glycerol-free TEY and incubated in a dry bath (37 °C/5 min). Subsequently, an aliquot of 2.5 μL of the sample was deposited on a slide, covered with a coverslip (18 x 18 mm), both previously heated (37 °C), and evaluated in the CASA system equipped with a phase contrast microscope (Eclipse 50i; Nikon, Japan). The images were captured with a Basler A312FC digital camera (Basler Vision Technologies, Germany), and the SCA™ software, version 5.1 (Microptics, S.L., Barcelona, Spain), and used to evaluate the kinematic parameters. For all analyses, five random fields were selected, with records of at least 500 sperm cells. The kinematic parameters evaluated were as follows: total motility (TM, %), progressive motility (PM, %), linearity (LIN, %), straightness (STR, %), curvilinear velocity (VCL, μm/s), straightline velocity (VSL, μm/s), average path velocity (VAP, μm/s), amplitude of lateral head displacement (ALH, μm), and beat frequency of the tail (BCF, Hz).

#### Plasma membrane integrity (PMi)

Plasma membrane integrity was determined by the double staining method, using DCF (C5141) and IP (P4170) fluorochromes, according to Silva et al. (2019). For each sample, an aliquot (10 μL) of semen was diluted in 30 μL of Tris solution, containing 5 μL DCF and 5 μL PI, and incubated for 10 min (25 °C). Using DBP 485/20-nm filters for excitation and DBP 580–630-nm ones for emission, 200 sperm cells per slide were analyzed under an epifluorescence microscope (Carl Zeiss, Göttingen, Germany) at a magnification of 400 X. Spermatozoa stained green were considered intact, and those stained red were considered to have a damaged membrane.

#### Acrosomal membrane integrity (ACi)

Evaluation of the acrosomal membrane integrity was performed using the fluorochrome FITC-PNA (Silva et al., 2019). For all treatments, an aliquot (10 μL) was used to make a smear, which was stained with 20 μL of FITC-PNA and incubated in a humid chamber (4 °C) for 15 min in the dark. Subsequently, all slides were immersed in PBS twice and air-dried. Immediately before the evaluation, 5.0 μL of UCD solution (4.5 mL of glycerol, 0.5 mL of PBS, and 5.0 mg of p-phenylenediamine) was placed on the slide, covered with a coverslip. Two hundred sperm cells were randomly counted under an epifluorescence microscope (Carl Zeiss; Germany, 1,000X), using an excitation filter at BP 450–490 nm and LP emission at 515 nm, and cells with an intact acrosome were considered to be those that had the acrosome stained fluorescent green. Sperm cells with reacted acrosomes were all those that had the equatorial region of the sperm head fluoresced in green or did not have fluorescence in the acrosome region.

#### Mitochondrial membrane potential (MMP)

The MMP was evaluated according to Silva et al. (2019), using the lipophilic cationic fluorochrome JC-1 (0.15 Mm in DMSO). For each group, a 10-μL aliquot of the sample was diluted in 30 μL of Tris buffer medium and then stained with 5 μL of JC-1 and incubated at 25 °C for 10 min. For analysis, sperm cells were evaluated under a fluorescence microscope (Carl Zeiss, Göttingen, Germany; 400 X), using an excitation filter of BP 450-490 nm and LP emission of 515 nm. Sperm cells with the middle piece stained in orange were classified as having a high mitochondrial membrane potential, and those with the middle piece stained in green were classified as having a low mitochondrial membrane potential.

### Statistical analysis

The semen samples were distributed in a completely randomized experimental design with a factorial scheme (4 × 2), using four concentrations (0.0%, 1.5%, 2.5%, and 3.5%) of two essential oils.

GraphPad InStat (version 3.10, 2009) was used for all statistical analyses. Data were evaluated for normality and homogeneity of variance using the Kolmogorov–Smirnov test. For each experimental group, the data were submitted to analysis of variance to determine treatment effects, with a significance level of 5%. When significant differences were found, the Tukey–Kramer test was performed to compare means. Results are expressed as mean ± standard deviation. Differences with a P value of ≤0.05 were considered statistically significant.

## Results

### Macroscopic physical analysis

The macroscopic physical analysis of the nanoemulsions containing essential oil from Sicilian lemon or wild orange was performed immediately (0 h) after preparation. The formulations showed a homogeneous, fluid, and opaque appearance, with no lumps or odor, but with color present. These characteristics remained unchanged after 24 hours of thermal stress (Table 2).

**Table 2:** Organoleptic characteristics of nanoemulsions using Sicilian lemon and wild orange essential oils, with soy lecithin as surfactant (5%), immediately after (0 h) or subjected to heat stress of cooling (5 ± 2 °C), freezing (−20 ± 2 °C), and heating (37, 40 and 50 ± 2 °C), for a period of 24 h after formulation.

**Evaluation time**	**Essential oil**	**Aspect**	**Consistency**	**Opacity**	**Lumps**	**Odor**	**Colour**
**0 h**	**Sicilian lemon**	HM	FL	ON	AT	AT	PR
	**Wild orange**	HM	FL	ON	AT	AT	PR
**24 h**	**Sicilian lemon**	HM	FL	ON	AT	AT	PR
	**Wild orange**	HM	FL	ON	AT	AT	PR

HM: Homogeneous; FL: Fluid; O: Opaque; AT: Absent; PR: Present.

### Physicochemical characterization

The physicochemical characterization of the nanoemulsions containing essential oil from Sicilian lemon or wild orange (Table 3) showed vesicles with average sizes below 220.00 nm, a PDI of <0.30, and a zeta potential of −59.00 mV.

**Table 3:** Physicochemical characterization of oil-in-water (O/A) essential oil nanoemulsions of Sicilian lemon and wild orange, using soy lecithin (5%) as surfactant.

**Nanoemulsion**	**Vescile Size (nm)**	**Polydispersity Index**	**Zeta potential (mv)**
**Sicilian Lemon**	219.63 ± 31.81	0.184	-59.00 ± 3.40
**Wild orange**	205.17 ± 17.06	0.211	-59.00 ± 3.96

### Kinematics and sperm integrity analysis

In the analysis of sperm kinematics, all groups showed total motility of ≥40% after thawing the semen samples supplemented with different concentrations of Sicilian lemon or wild orange essential oil nanoemulsion (Table 4). However, no significant differences (P > 0.05) were observed among the groups (0.0%, 1.5%, 2.5%, and 3.5%), regardless of the oil used, across all kinematic parameters of ram spermatozoa subjected to freezing.

**Table 4:** Kinematic parameters and percentages of thawed ram sperm cells with intact plasma membranes (plasma membrane integrity [PMi]), intact acrosomes (acrosomal membrane integrity [ACi]), and high mitochondrial membrane potential (MMP), cryopreserved in Tris–egg yolk extender supplemented with different concentrations of Sicilian lemon (*Citrus limon*) or wild orange (*Citrus sinensis*) essential oil nanoemulsions and using soy lecithin (5%) as a surfactant. Data are expressed as mean ± standard deviation.

**Sperm parameter**	**Sicilian lemon essential oil nanoemulsion**				**Wild orange essential oil nanoemulsion**			
	**0.0%**	**1.5%**	**2.5%**	**3.5%**	**0.0%**	**1.5%**	**2.5%**	**3.5%**
TM (%)	50.04±8.51	53.83±8.56	49.67±7.37	55.47±6.48	50.04±8.51	51.56±4.14	54.34±6.92	47.93±6.05
PM (%)	22.26±7.41	25.11±2.63	26.89±8.39	26.24±7.35	22.26±7.41	26.71±5.50	28.23±8.52	26.39±5.97
VCL (µm/s)	75.46±4.19	75.37±6.75	78.57±10.74	74.44±7.65	75.46±4.19	78.89±13.09	76.50±8.72	79.41±8.48
VSL (µm/s)	46.93±7.08	48.39±4.84	53.71±11.68	48.50±5.29	46.93±7.08	52.96±10.15	50.96±11.05	54.73±10.42
VAP (µm/s)	59.77±5.70	61.21±6.46	64.83±11.58	60.63±5.12	59.77±5.70	64.89±11.38	62.53±10.41	66.34±10.03
LIN (%)	62.24±8.52	64.35±4.46	67.99±7.88	65.59±8.42	62.24±8.52	66.18±4.33	68.03±6.01	69.00±8.49
STR (%)	78.41±7.77	79.24±4.66	82.53±4.86	80.10±7.12	78.41±7.77	81.47±4.59	80.96±5.36	82.10±4.87
ALH (µm)	2.20±0.27	2.26±0.24	2.21±0.31	2.16±0.34	2.20±0.27	2.26±0.34	2.20±0.31	2.14±0.19
BCF (Hz)	10.43±1.39	9.69±0.85	9.80±1.08	9.46±1.51	10.43±1.39	10.03±1.55	10.07±1.66	9.44±1.24
PMi (%)	32.50±2.12	34.43±3.35	32.36±3.60	33.36±10.55	32.50±2.12	36.07±9.29	37.43±4.47	31.71±4.42
ACi (%)	42.50±3.97^b^	47.71±6.92^ab^	51.43±6.95^a^	49.79±7.01^a^	42.50±3.97^b^	51.43±6.53^a^	52.64±6.22^a^	58.71±4.61^a^
MMP (%)	47.50±10.59	47.29±7.45	45.00±3.55	44.07±8.24	47.50±10.59	50.64±9.78	46.57±6.70	45.64±6.50

TM: total motility; PM: progressive motility; VCL: curvilinear velocity; VSL: straightline velocity; VAP: average path velocity; LIN: linearity; STR: straightness; ALH: amplitude of lateral head displacement; BCF: beat frequency of the tail. Different lowercase letters, when present, on the same line, indicate a significant difference (P<0.05) between the groups in the same nanoemulsion (Sicilian lemon or Wild orange).

Epifluorescence microscopy analysis (Table 4) showed that supplementation of the Tris–egg yolk diluent with Sicilian lemon or wild orange nanoemulsion during the freezing of ram semen did not result in significant differences (P > 0.05) in the percentage of spermatozoa with intact plasma membranes and high mitochondrial membrane potential post-thawing, regardless of the concentration used (1.5%, 2.5%, and 3.5%) when compared with the control group. However, the percentages of spermatozoa with intact acrosome membranes were higher (P > 0.05) in samples frozen in Tris–egg yolk extender supplemented with lemon essential oil nanoemulsion at concentrations of 2.5% and 3.5% compared with the control. Similarly, semen samples frozen with wild orange essential oil nanoemulsion at concentrations of 1.5%, 2.5%, and 3.5% also showed higher percentages (P > 0.05) of spermatozoa with intact acrosomes relative to the control group.

## Discussion

Studies have been conducted using nanoemulsions of essential oils to enhance their solubility, bioavailability, and functionality (Khalil et al., 2023). This is the first study to evaluate the influence of nanoemulsions of Sicilian lemon and wild orange essential oils, used as additives in a Tris–egg yolk extender for ram semen freezing, on post-thaw sperm cell viability. This work expands our understanding of applying nanotechnology to semen cryopreservation—specifically, the supplementation of a freezing extender with essential oil nanoemulsions of Sicilian lemon and wild orange, using soy lecithin as a surfactant.

After preparation, the organoleptic characteristics of the nanoemulsions (Sicilian lemon and wild orange) were found to be homogeneous, fluid, opaque, colored, lump-free, and odorless. Meanwhile, physicochemical analysis revealed vesicles with nanometric size (<220.0 nm), which is considered a favorable characteristic in a nanostructured system. According to Preeti et al. (2023), the smaller the vesicle size, the greater the spreadability and transport capacity in the medium. It should also be noted that despite being thermodynamically unstable systems, the small vesicles obtained in this study may indicate greater stability and retention capacity. This allows for a longer period of residence in the cells (Singh et al., 2017).

The PDI of the nanoemulsions (Sicilian lemon and wild orange), which refers to the homogeneity in vesicle size, presented values below 0.30—an indication of uniformity among the vesicles (Maherani and Wattraint, 2017). This is important because the vesicle size distribution influences both the properties of nanostructured systems (such as stability, volume, and appearance) and the vesicles absorption capacity by cells (Danaei et al., 2018). The PDI values obtained in this study (<0.30) are consistent with findings by Badran (2014) and Karimirad et al. (2020), who reported that PDI values below 0.30 and 0.45, respectively, indicate homogeneity in vesicle size and higher quality of the systems. By contrast, a PDI of >0.70 suggests a wide granulometric distribution (Shah et al., 2015; Danaei et al., 2018). Finally, the nanoemulsions produced in this study showed a size (<200 nm) and PDI (<0.30) on a scale comparable to that developed by Donsí et al. (2012), who also used soy lecithin (3%) as a surfactant and obtained vesicles with sizes of <250 nm and PDI of <0.40. This suggests that the nanoemulsions produced here are of comparable quality to those in previous research, indicating a successful formulation process.

Zeta potential is an indicator of colloidal stability influenced by surface charge (Khalil et al., 2019). Nanoemulsions consist of electrically charged particles, and the magnitude of the zeta potential determines the stability of interactions between particles and between the particles and the surrounding medium (Midekessa et al., 2020). In this study, the nanoemulsions produced exhibited high negative charges (−59.00 mV), indicating good stability and aligning with the findings of Kaur et al. (2020), who reported −14.9 mV in nanoemulsions prepared with citrus essential oils. For optimal stability, systems should exhibit particles with either high negative or high positive zeta potential values because this promotes particle repulsion. By contrast, low zeta potential values reduce repulsion and increase the risk of aggregation (Khalil et al., 2019). Additionally, the negative zeta potential values observed in this study may suggest better storage stability (Rodrigues et al., 2018). This is because high absolute zeta potential values generate repulsive forces that exceed attractive forces, resulting in a stable system (Nasiri-Foomani et al., 2024) and minimizing coalescence of the dispersed phase (McClements, 2012). Coalescence occurs when vesicles fuse to form larger structures, directly impacting the overall stability of the nanoemulsions (Choi and McClements, 2020).

Few studies have explored the use of nanoemulsions in the preservation of sperm cells. Sánchez-Rúbio et al. (2020) demonstrated the efficacy of vitamin E nanoemulsions in preserving deer sperm cells, as they enhanced the antioxidant’s beneficial properties while reducing harmful effects on sperm. Based on these findings, the authors emphasized the importance of combining antioxidants with various nanosystems to improve performance in combating oxidative stress. Given that cell membranes act as selective barriers that regulate the entry and exit of cellular constituents (Moghadam et al., 2012), the nanometric size and homogeneity of the particles promote greater penetration into the cell (Acosta, 2009). They also enable better interaction with membrane components, facilitating entry into the intracellular environment (Najafi et al., 2019).

Considering that the formulation of the nanoemulsions (Sicilian lemon and wild orange) presented desirable nanometric characteristics—such as stability, vesicle size, PDI, and zeta potential—we expected that their addition at different concentrations (1.5%, 2.5%, and 3.5%) would have a positive effect on ovine sperm kinematics after thawing. However, no significant impacts were observed on sperm kinematics following the addition of nanoemulsions (Sicilian lemon and wild orange) post-thawing. These findings partially corroborate those of Branco et al. (2016a), who evaluated the influence of directly adding limonene (S)-(−) at concentrations of 50, 100, and 150 μM to a Tris–egg yolk freezing extender for bull semen. According to their results, the addition of 150 μM of limonene (S)-(−) resulted in significantly higher (P < 0.05) sperm linearity compared with the control and the other concentrations (50 and 100 μM), although it did not affect other kinematic parameters. These data suggest that differences in species (bovine vs. ram) and the type of supplementation (direct addition of limonene (S)-(−) vs. nanoemulsions of citrus essential oils rich in limonene) may explain the variations observed in sperm linearity. Furthermore, it is possible to hypothesize that the specific limonene content—64.96% in Sicilian lemon and 93.77% in wild orange—may account for the divergence in sperm linearity results found in this study when compared with those reported by Branco et al. (2016a).

The use of a computer-assisted sperm analysis system enables the assessment of sperm kinematics (Van der Horst, 2020), providing detailed information on various characteristics of sperm movement (Amann and Katz, 2004; Hidalgo et al., 2021). Other studies have correlated kinematic parameters with sperm viability and fertilizing capacity after thawing (Yánez-Ortiz et al., 2022). However, aside from motility—which is considered the most important—specific parameters directly related to fertility have not yet been clearly identified (Tsakmakidis, 2010). In the present study, the addition of essential oil nanoemulsions from Sicilian lemon and wild orange did not alter sperm motility or kinematic parameters. Nonetheless, total motility remained above the standard recommended by CBRA (2013) for artificial insemination of ewes using frozen/thawed semen.

The plasma membrane integrity and mitochondrial membrane potential observed in this study showed a pattern similar to that of sperm kinematics. These results are in agreement with those reported by Branco et al. (2016b), who directly used limonene (R)-(+) as an additive in the cryopreservation extender for bull semen to evaluate its influence on plasma membrane integrity and mitochondrial membrane potential post-thawing. The authors concluded that the addition of limonene (R)-(+) did not affect any of the evaluated parameters.

Plasma membrane integrity is related to various physiological processes and the survivability of sperm cells within the female genital tract (Gwathmey et al., 2006). This integrity is closely associated with cell viability (Chelucci et al., 2015), and the ability to maintain membrane permeability is a critical factor in overall sperm functionality (Peña et al., 2005).

Ram spermatozoa, in particular, are more sensitive to the cryopreservation process than are spermatozoa of other domestic ruminants. This increased sensitivity is attributed to the higher content of polyunsaturated phospholipids in the sperm membrane, which heightens fragility and makes the cells more susceptible to the action of reactive oxygen species (ROS) (Peris-Frau et al., 2020; Vozaf et al., 2022).

Notably, cryopreservation can lead to increased ROS production, a consequence of semen dilution and temperature fluctuations. This causes damage to sperm cells, resulting in an oxidant/antioxidant imbalance, reduced cellular activity and viability, and decreased motility, mitochondrial function, and plasma membrane integrity (Peris-Frau et al., 2020).

In this study, the negative effects of ROS on sperm membranes may have been minimized by the use of Sicilian lemon and wild orange essential oils. These oils contain high concentrations of limonene—64.96% and 93.77%, respectively—which is known for various therapeutic properties, particularly its antioxidant activity. Additionally, studies have linked limonene’s functionality to the inhibition of lipid peroxidation, helping to prevent cell damage caused by oxidants (Rehman et al., 2014), as well as to the elimination of these compounds and the interruption of the lipid oxidation cycle (Wei and Shibamoto, 2007). In light of this, research has intensified in recent years to further understand the antioxidant activity of limonene.

No effects were observed on plasma or mitochondrial membranes. Nevertheless, the analysis of acrosomal membrane integrity revealed that supplementing the freezing extender (Tris–egg yolk) with nanoemulsions of essential oils from Sicilian lemon (2.5% and 3.5%) and wild orange (1.5%, 2.5%, and 3.5%) resulted in higher percentages of spermatozoa with intact acrosomes after thawing. The fact that the lower concentration of Sicilian lemon (1.5%) did not differ from the control group—unlike wild orange at the same concentration—may be related to the lower limonene content in Sicilian lemon essential oil (64.96%) compared with that of wild orange (93.77%).

The results regarding acrosome integrity suggest a beneficial effect of nanoemulsions of Sicilian lemon (2.5% and 3.5%) and wild orange (1.5%, 2.5%, and 3.5%) on the fertilizing capacity of these gametes. This is significant because the integrity of the acrosome is crucial to the fertilization process (Sun et al., 2020), being directly related to the sperm cell’s ability to bind to the zona pellucida (Cui et al., 2000).

The ability to increase the number of cells with intact acrosomes may be attributed to the composition of the nanoemulsions. Limonene is the main constituent of the essential oils (Sicilian lemon and wild orange), and soy lecithin is used as a surfactant in the formulation. Soy lecithin is composed of a mixture of phosphatidylcholine, phosphatidylethanolamine, and phosphatidylinositol (Mertins et al., 2008), along with stearic, oleic, and palmitic fatty acids (Kumar et al., 2015), which are also present in biological membranes and contribute to their stability (Crespilho et al., 2012). According to Chelucci et al. (2015), lecithin is a viable alternative as a component of semen cryopreservation extenders. However, these authors also note that the mechanism by which lecithin protects sperm cell membranes during the freezing/thawing process has not yet been fully elucidated.

Zhang et al. (2009) suggested that the phospholipids in soy lecithin do not penetrate the plasma membrane but instead form a protective envelope around the sperm cell. This minimizes the formation of intracellular ice crystals and maintains membrane integrity during the freeze/thaw process. However, another possibility related to our results is the presentation of soy lecithin at the nanometric scale, which may allow greater interaction with the plasma membrane. Nadri et al. (2019) evaluated the influence of lecithin, egg yolk, and lecithin nanoparticle-based extenders on goat sperm cells after thawing. They concluded that a nanolecithin-based extender at a concentration of 2% can improve the cryosurvival of goat spermatozoa.

In this study, the nanometer-sized presentation of lecithin may have enhanced its effects due to a higher surface-to-volume ratio. This facilitates the interaction of nanolecithin with sperm cells (Nadri et al., 2019) and contributes to improved acrosomal membrane integrity. According to Tadros et al. (2004), the small vesicle size of nanostructured systems promotes better spreadability and facilitates penetration and absorption of constituents (Irache et al., 2011). Supporting this hypothesis, Sun et al. (2021) observed that the addition of soy lecithin nanoparticles (0.5% and 1.0%) to the freezing extender (modified Lake) for rooster semen improved the acrosome integrity of sperm cells.

Thus, this study expands our understanding of nanotechnology as it relates to the cryopreservation of ram semen—specifically, the use of nanoemulsions of essential oils from Sicilian lemon and wild orange, with soy lecithin (5%) as a surfactant, as additives to the freezing extender and their effects on sperm cell viability after thawing. Based on the results obtained, it is clear that further *in vivo* studies are needed to better understand the mechanisms by which nanoemulsions influence sperm viability.

## Conclusion

Nanoemulsions of essential oils from Sicilian lemon (2.5% and 3.5%) and wild orange (1.5%, 2.5%, and 3.5%), formulated with soy lecithin (5.0%) as a surfactant, can be used as additives to the Tris–egg yolk extender for ram semen freezing. These nanoemulsions help preserve acrosome integrity post-thawing.

## Data Availability

Research data is only available upon request.

## References

[B001] Acosta E (2009). Bioavailability of nanoparticles in nutrient and nutraceutical delivery. Curr Opin Colloid Interface Sci.

[B002] Amann RP, Katz DF (2004). Reflections on CASA after 25 years. J Androl.

[B003] Anandakumar P, Kamaraj S, Vanitha MK (2021). D-limonene: A multifunctional compound with potent therapeutic effects. J Food Biochem.

[B004] ANVISA (2004). Guia de estabilidade de produtos cosméticos..

[B005] Arando A, Gonzalez A, Delgado JV, Arrebola FA, Perez-Marín CC (2017). Storage temperature and sucrose concentrations affect ram sperm quality after vitrification. Anim Reprod Sci.

[B006] Badran M (2014). Formulation and in vitro evaluation of flufenamic acid loaded deformable liposome for improved skin delivery. Dig J Nanomater Biostruct.

[B007] Branco YNTCC, Branco MAC, Gomes LA, Lustosa MSC, Barçante FPS, Barros FN, Nascimento IMR, Souza JAT (2016). Ação do Limoneno (S)-(-). sobre a cinética espermática de sêmen criopreservado de touros. Rev Bras Reprod Anim.

[B008] Branco YNTCC, Branco MAC, Souza A, Rodrigues VS, Moraes FJ, Evangelista LSM, Nascimento IMR, Souza JAT (2016). Avaliação da integridade da membrana plasmática e do potencial de atividade mitocondrial de sêmen criopreservado de touros frente a ação do (Limoneno (R)-. Rev Bras Reprod Anim.

[B009] Cadena PG, Pereira MA, Cordeiro RBS, Cavalcanti IMF, Barros B, Pimentel MCCB, Lima JL, Silva VL, Santos-Magalhães NS (2013). Nanoencapsulation of quercetin and resveratrol into elastic liposomes. Biochim Biophys Acta.

[B010] CBRA (2013). Manual de exame andrológico e avaliação de sêmen animal..

[B011] Chelucci S, Pasciu V, Succu S, Addis D, Leoni GG, Manca ME, Naitana S, Berlinguer F (2015). Soybean lecithin–based extender preserves spermatozoa membrane integrity and fertilizing potential during goat semen cryopreservation. Theriogenology.

[B012] Choi SJ, McClements DJ (2020). Nanoemulsions as delivery systems for lipophilic nutraceuticals: strategies for improving their formulation, stability, functionality and bioavailability. Food Sci Biotechnol.

[B013] Crespilho AM, Sá MF, Dell’Aqua JA, Nichi M, Monteiro GA, Avanzi BR, Martins A, Papa FO (2012). Comparison of in vitro and in vivo fertilizing potential of bovine semen frozen in egg yolk or new lecithin based extenders. Livest Sci.

[B014] Cui YH, Zhao RL, Wang Q, Zhang ZY (2000). Determination of sperm acrosin activity for evaluation of male fertility. Asian J Androl.

[B015] Damasceno GAB, Silva RMAC, Fernandes JM, Ostrosky EA, Langassner SMZ, Ferrari M (2016). Use of *Opuntia ficus-indica* (*L.*) Mill extracts from Brazilian Caatinga as an alternative of natural moisturizer in cosmetic formulations. Braz J Pharm Sci.

[B016] Danaei M, Dehghankhold M, Ataei S, Hasanzadeh Davarani F, Javanmard R, Dokhani A, Khorasani S, Mozafari MR (2018). Impact of particle size and polydispersity index on the clinical applications of lipidic nanocarrier. Systems Pharm..

[B017] Davicino R, Micucci P, Sebastian T, Graciela F, Anesini C (2009). Antioxidant activity of limonene on normal murine lymphocytes: relation to H_2_O_2_ modulation and cell proliferation. Basic Clin Pharmacol Toxicol.

[B018] Donsì F, Annunziata M, Vincensi M, Ferrari G (2012). Design of nanoemulsion-based delivery systems of natural antimicrobials: effect of the emulsifier. J Biotechnol.

[B019] Fisher K, Phillips C (2008). Potential antimicrobial uses of essential oils in food: is citrus the answer?. Trends Food Sci Technol.

[B020] Gwathmey TM, Ignotz GG, Mueller JL, Manjunath P, Suarez SS (2006). Bovine seminal plasma proteins PDC-109, BSP-A3, and BSP-30-kDa share functional roles in storing sperm in the oviduct. Biol Reprod.

[B021] Hidalgo MMT, Almeida ABM, Moraes FLZ, Marubayashi RYP, Souza FF, Barreiros TRR, Martins MIM (2021). Sperm subpopulations influence the pregnancy rates in cattle. Reprod Domest Anim.

[B022] Hulla JE, Sahu SC, Hayes AW (2015). Nanotechnology: history and future. Hum Exp Toxicol.

[B023] Irache JM, Esparza I, Gamazo C, Agüeros M, Espuelas S (2011). Nanomedicine: novel approaches in human and veterinary therapeutics. Vet Parasitol.

[B024] Karimirad R, Behnamian M, Dezhsetan S (2020). Bitter orange oil incorporated into chitosan nanoparticles: Preparation, characterization and their potential application on antioxidant and antimicrobial characteristics of white button mushroom. Food Hydrocoll.

[B025] Kaur H, Pancham P, Kaur R, Agarwal S, Singh M (2020). Synthesis and characterization of Citrus limonum essential oil based nanoemulsion and its enhanced antioxidant activity with stability for transdermal application. J Biomater Nanobiotechnol.

[B026] Khalil WA, El-Harairy MA, Zeidan AEB, Hassan MAE (2019). Impact of selenium nano-particles in semen extender on bull sperm quality after cryopreservation. Theriogenology.

[B027] Khalil WA, Hassan MAE, Attia KAA, El-Metwaly HÀ, El-Harairy MA, Sakr AM, Abdelnour AS (2023). Effect of olive, flaxseed, and grape seed nano-emulsion essential oils on semen buffalo freezability. Theriogenology.

[B028] Kumar P, Saini M, Kumar D, Balhara AK, Yadav SP, Singh P, Yadav OS (2015). Liposome-based semen extender is suitable alternative to egg yolk-based extender for cryopreservation of buffalo (*Bubalus bubalis*) semen. Anim Reprod Sci.

[B029] Lv C, Wu G, Hong Q, Quan G (2019). Spermatozoa cryopreservation: state of art and future in small ruminants. Biopreserv Biobank.

[B030] Maherani B, Wattraint O (2017). Liposomal structure: a comparative study on light scattering and chromatography techniques. J Dispers Sci Technol.

[B031] Mcclements D (2012). Nanoemulsions versus microemulsions: terminology, differences, and similarities. Soft Matter.

[B032] Mehl F, Marti G, Boccard J, Debrus B, Merle P, Delort E, Baroux L, Raymo V, Velazco MI, Sommer H, Wolfender JL, Rudaz S (2014). Differentiation of lemon essential oil based on volatile and nonvolatile fractions with various analytical techniques: a metabolomic approach. Food Chem.

[B033] Mertins O, Sebben M, Schneider PH, Pohlmann AR, Silveira NP (2008). Caracterização da pureza de fosfatidilcolina da soja através de RMN de ^1^ H e de ^31^P. Quim Nova.

[B034] Midekessa G, Godakumara K, Ord J, Viil J, Lättekivi F, Dissanayake K, Kopanchuk S, Rinken A, Andronowska A, Bhattacharjee S, Rinken T, Fazeli A (2020). Zeta potential of extracellular vesicles: toward understanding the attributes that determine colloidal stability. ACS Omega.

[B035] Moghadam BY, Hou WC, Corredor C, Westerhoff P, Posner JD (2012). Role of nanoparticle surface functionality in the disruption of model cell membranes. Langmuir.

[B036] Mokhtassi-Bidgoli A, Sharafi M, Benson JD (2023). Optimizing Bull semen cryopreservation media using multivariate statistics approaches. Animals.

[B037] Nadri T, Towhidi A, Zeinoaldini S, Martínez-Pastor F, Mousavi M, Noei R, Tar M, Mohammadi Sangcheshmeh A (2019). Lecithin nanoparticles enhance the cryosurvival of caprine sperm. Theriogenology.

[B038] Najafi A, Kia HD, Hamishehkar H, Moghaddam G, Alijani S (2019). Effect of resveratrol-loaded nanostructured lipid carriers supplementation in cryopreservation medium on post-thawed sperm quality and fertility of roosters. Anim Reprod Sci.

[B039] Nasiri-Foomani N, Ebadi M, Hassani S, Zeinoaldini S, Saedi A, Samadi F (2024). Preparation, characterization, and ex-vivo evaluation of curcumin-loaded niosomal nanoparticles on the equine sperm quality during cooled storage. Int J Biol Macromol.

[B040] Osanloo M, Ghaznavi G, Abdollahi A (2020). Surveying the chemical composition and antibacterial activity of essential oils from selected medicinal plants against human pathogens. Iran J Microbiol.

[B041] Palazzolo E, Laudicina VA, Germanà MA (2013). Current and potential use of citrus essential oils. Curr Org Chem.

[B042] Peña FJ, Saravia F, Johannisson A, Walgren M, Rodríguez-Martínez H (2005). A new and simple method to evaluate early membrane changes in frozen–thawed boar spermatozo. Int J Androl.

[B043] Peris-Frau P, Martín-Maestro A, Iniesta-Cuerda M, Sánchez-Ajofrín I, Cesari AJ, Garde J, Villar M, Soler AJ (2020). Cryopreservation of ram sperm alters the dynamic changes associated with in vitro capacitation. Theriogenology.

[B044] Preeti SS, Malik R, Bhatia S, Al Harrasi A, Rani C, Saharan R, Kumar S, Geeta SR, Sehrawat R (2023). Nanoemulsion: an emerging novel technology for improving the bioavailability of drugs. Scientifica.

[B045] Rehman MU, Tahir M, Khan AQ, Khan R, Oday-O-Hamiza A, Lateef A, Hassan SK, Rashid S, Ali N, Zeeshan M, Sultana S (2014). D-limonene suppresses doxorubicin-induced oxidative stress and inflammation via repression of COX-2, iNOS, and NFκB in kidneys of Wistar rats. Exp Biol Med.

[B046] Rodrigues FVS, Diniz LS, Sousa RMG, Honorato TD, Simão DO, Araújo CRM, Gonçalves TM, Rolim LA, Goto PL, Tedesco AC, Siqueira-Moura MP (2018). Preparation and characterization of nanoemulsion containing a natural Naphthoquinone. Quim Nova.

[B047] Sánchez-Rubio F, Soria-Meneses PJ, Jurado-Campos A, Bartolomé-García J, Gómez-Rubio V, Soler AJ, Arroyo-Jimenez MM, Santander-Ortega MJ, Plaza-Oliver M, Lozano MV, Garde JJ, Fernández-Santos MR (2020). Nanotechnology in reproduction: vitamin E nanoemulsions for reducing oxidative stress in sperm cells. Free Radic Biol Med.

[B048] Santos FR, Souza JLC, Silva BR, Pereira CO, Flores RA, Souza ERB (2023). Yield of essential oil from varieties of Citrus sinensis (L.) Osbeck. Rev Bras Frutic.

[B049] Santos-Magnabosco AR, Quinova EID, Melo MVV, Bastos PES, Santos TP, Silva II, Andrade ALC, Padilha RMO, Silva JF, Sá FB, Cadena MRS, Cadena PG (2022). Testosterone nanoemulsion produced masculinized Nile tilapia (*Oreochromis niloticus*). Fish Physiol Biochem.

[B050] Shah R, Eldridge D, Palombo E, Harding I (2015). Lipid nanoparticles: production, characterization and stability..

[B051] Silva RAJA, Batista AM, Arruda LCP, Souza HM, Nery IHAV, Gomes WA, Soares PC, Silva SV, Guerra MMP (2019). Concentration of soybean lecithin affects short-term storage success of goat semen related with seminal plasma removal. Anim Reprod.

[B052] Singh Y, Meher JG, Raval K, Khan FA, Chaurasia M, Jain NK, Chourasia MK (2017). Nanoemulsion: concepts, development and applications in drug delivery. J Control Release.

[B053] Sitarek P, Rijo P, Garcia C, Skała E, Kalemba D, Białas AJ, Szemraj J, Pytel D, Toma M, Wysokińska H, Śliwiński T (2017). Antibacterial, Anti-Inflammatory, Antioxidant, and Antiproliferative properties of essential oils from hairy and normal roots of *Leonurus sibiricus* L. and their chemical composition. Oxid Med Cell Longev.

[B054] Sun L, He M, Wu C, Zhang S, Dai J, Zhang D (2021). Beneficial influence of soybean lecithin nanoparticles on rooster frozen–thawed semen quality and fertility. Animals.

[B055] Sun W, Jiang S, Su J, Zhang J, Bao X, Ding R, Shi P, Li S, Wu C, Zhao G, Cao G, Sun QY, Yu H, Li X (2020). The effects of cryopreservation on the acrosome structure, enzyme activity, motility, and fertility of bovine, ovine, and goat sperm. Anim Reprod.

[B056] Tadros T, Izquierdo P, Esquena J, Solans C (2004). Formation and stability of nano-emulsions. Adv Colloid Interface Sci.

[B057] Tsakmakidis IA (2010). Ram semen evaluation: development and efficiency of modern techniques. Small Rumin Res.

[B058] Van der Horst G (2020). Computer Aided Sperm Analysis (CASA) in domestic animals: current status, three D tracking and flagellar analysis. Anim Reprod Sci.

[B059] Vozaf J, Svoradová A, Baláži A, Vašíček J, Olexiková L, Dujíčková L, Makarevich AV, Jurčík R, Ďúranová H, Chrenek P (2022). The cryopreserved sperm traits of various ram breeds: towards biodiversity conservation. Animals.

[B060] Wei A, Shibamoto T (2007). Antioxidant activities and volatile constituents of various essential oils. J Agric Food Chem.

[B061] Yánez-Ortiz I, Catalán J, Rodríguez-Gil JE, Miró J, Yeste M (2022). Advances in sperm cryopreservation in farm animals: cattle, horse, pig and sheep. Anim Reprod Sci.

[B062] Yeste M (2017). State-of-the-art of boar sperm preservation in liquid and frozen state. Anim Reprod.

[B063] Zhang S, Hu J, Li Q, Jiang Z, Zhang X (2009). The cryoprotective effects of soybean lecithin on boar spermatozoa quality. Afr J Biotechnol.

